# A Genetic Risk Score Is Associated with Weight Loss Following Roux-en Y Gastric Bypass Surgery

**DOI:** 10.1007/s11695-016-2072-9

**Published:** 2016-01-30

**Authors:** Marcus Bandstein, Sarah Voisin, Emil K. Nilsson, Bernd Schultes, Barbara Ernst, Martin Thurnheer, Christian Benedict, Jessica Mwinyi, Helgi B. Schiöth

**Affiliations:** 1 Department of Neuroscience, Functional Pharmacology, Uppsala University, Box 593, 751 24 Uppsala, Sweden; 2 Interdisciplinary Obesity Center, eSwiss Medical & Surgical Center, St. Gallen, Switzerland

**Keywords:** Genetic risk score, Roux-en Y gastric bypass surgery, Obesity, Random forest model, Post-operative weight loss

## Abstract

**Background:**

Currently, Roux-en Y gastric bypass (RYGB) is the most efficient therapy for severe obesity. Weight loss after surgery is, however, highly variable and genetically influenced. Genome-wide association studies have identified several single nucleotide polymorphisms (SNP) associated with body mass index (BMI) and waist-hip ratio (WHR). We aimed to identify two genetic risk scores (GRS) composed of weighted BMI and WHR-associated SNPs to estimate their impact on excess BMI loss (EBMIL) after RYGB surgery.

**Methods:**

Two hundred and thirty-eight obese patients (BMI 45.1 ± 6.2 kg/m^2^, 74 % women), who underwent RYGB, were genotyped for 35 BMI and WHR-associated SNPs and were followed up after 2 years. SNPs with high impact on post-surgical weight loss were filtered out using a random forest model. The filtered SNPs were combined into a GRS and analyzed in a linear regression model.

**Results:**

An up to 11 % lower EBMIL with higher risk score was estimated for two GRS models (*P* = 0.026 resp. *P* = 0.021) composed of seven BMI-associated SNPs (closest genes: *MC4R*, *TMEM160*, *PTBP2*, *NUDT3*, *TFAP2B*, *ZNF608*, *MAP2K5*, *GNPDA2*, and *MTCH2*) and of three WHR-associated SNPs (closest genes: *HOXC13*, *LYPLAL1*, and *DNM3*-*PIGC*). Patients within the lowest GRS quartile had higher EBMIL compared to patients within the other three quartiles in both models.

**Conclusions:**

We identified two GRSs composed of BMI and WHR-associated SNPs with significant impact on weight loss after RYGB surgery using random forest analysis as a SNP selection tool. The GRS may be useful to pre-surgically evaluate the risks for patients undergoing RYGB surgery.

**Electronic supplementary material:**

The online version of this article (doi:10.1007/s11695-016-2072-9) contains supplementary material, which is available to authorized users.

## Introduction

While obesity is today one of the world leading health problems, treatment options for obese patients remain limited. Roux-en Y gastric bypass (RYGB) surgery is currently one of the most efficient therapy alternatives for sustainable weight loss in obese patients [1]. However, the degree of weight loss and treatment success is highly variable and potential surgery-associated complications should not be underestimated [2–4]. Thus, the detection of reliable pre-surgical indicators for a risk-benefit assessment before the intervention would be of high value. Weight loss following RYGB surgery is influenced by genetic variants [5]. Large genome-wide association studies (GWAS) have detected several single nucleotide polymorphisms (SNP) that are associated with BMI and waist-hip ratio (WHR) [6–8]. It cannot be excluded that the genetic variants factors associated with BMI may have an influence on Roux-en Y surgery induced weight loss.

The *fat mass and obesity associated gene* (*FTO*) has the strongest association to BMI as shown in a study including more than 340,000 people [7]. However, the explained variance of the *FTO* effect allele was earlier published to be only 0.34 % [8], meaning that the remaining individual variance in BMI may be attributed to other genetic and non-genetic factors. Genetic risk scores (GRS) describe the collective impact of several potentially risk contributing SNPs by creating one continuous variable that indicates the likelihood to develop a disease or a trait, such as weight loss [9]. GRSs are calculated in a weighted or unweighted manner. While weighted GRS take the previously reported effect from of each individual SNP into account, unweighted GRS consider only the number of alleles of each SNP included in the calculation [10–12]. In the present study, we investigated the impact of BMI and WHR-associated SNPs on excess BMI loss (EBMIL) 2 years after RYGB surgery. Based on a selection of genetic variants, we developed a weighted GRS and estimated the post-surgery weight loss in dependency of the considered risk variants. To select SNPs having a relevant impact on EBMIL, the random forest statistical method was used [13]. This machine learning tool has been used previously to screen or reduce dimensions in large GWAS datasets [14–17]. In the current study, this statistical method is used for the first time to analyze and evaluate polymorphisms associated with weight loss after bariatric surgery.

## Material and Methods

### Patients

Two hundred and thirty-eight obese patients, characterized by a BMI >30 kg/m^2^, were included in the study (mean ± SD; age 43.2 ± 10.8, initial BMI 45.1 ± 6.2 kg/m^2^, 74 % women). All patients were weighted in the morning in a fasted state with light clothes but no shoes on. All participants underwent proximal or distal RYGB surgery at the Interdisciplinary Obesity Center, St. Gallen, Switzerland, and returned 2 years later to the study center for a follow-up investigation. Weight loss after surgery was determined as EBMIL, considering a BMI >25 kg/m^2^ as excess. The relative EBMIL was calculated as 100 − [(final BMI − 25/initial BMI − 25) * 100] [18]. Demographic and clinical characteristics of the study population are summarized in Table 1. Percent BMI loss was calculated according to [18] and was used as a confirmatory independent variable in genetic association analyses.

**Table 1:** Demographic and clinical characteristics

Variable	
Included patients, *n*	238
Sex, *n* (% cohort)	
Female	173 (73)
Male	65 (27)
Age^a^, years (SD)	43.1 (±10.8)
BMI^a^, kg/m^2^ (SD)	45.1 (±6.2)
Surgery type, *n* (% followed up cohort)	
Distal RYGB	175 (73.5)
Proximal RYGB	63 (26.5)
BMI^b^, kg/m^2^ (SD)	28.5 (±3.9)
EBMIL^b^, % (SD)	83.8 (±17.8)
BMI loss^b^, % (SD)	36.3 (±8.4)

*EBMIL* excess BMI loss, *RYGB* Roux-en Y gastric bypass

^a^At baseline

^b^At 24-month post-Roux-en Y gastric bypass surgery

The cohort and both bariatric procedures have earlier been described in detail [19]. In brief, a gastric pouch of about 20–30 ml was created after removing a major part of the stomach. The length to the biliopancreatic limb was set to approximately 60 cm in the proximal RYGB procedure and 60 to 100 cm in the distal procedure. Patients who obtained the operation for a second time or underwent an alternative intervention to RYGB surgery (e.g., gastric banding) were not included.

### Genotyping

Genotyping was performed by the SNP&SEQ Technology Platform at SciLifeLab, Uppsala, Sweden, using the Illumina iSelect genotyping array (Illumina Inc.). All determined SNPs were in Hardy-Weinberg equilibrium. None of the variants were in linkage disequilibrium. Twenty-three BMI-associated and 12 WHR-associated SNPs were chosen for genotyping out of large GWAS, reporting the SNPs’ association to BMI, respectively, WHR [6, 8]. Further, a minor allele frequency of >15 % was required. The full list of determined genetic variants is presented in Supplementary table 1.

### Statistics

The genotype of each SNP was coded based on the amount of effect alleles, i.e., 0 for no effect alleles, one for heterozygote carriers, and two for individuals carrying two effect alleles. Subsequently, SNP-associated beta-values as published by Speliotes et al. and Heid et al. were multiplied with the amount of alleles to obtain weighted SNP scores. Weighted scores were included in the random forest model [13] as predictors and EBMIL as the target variable. Ten thousand random decision trees were created. The random forest model result for the weighted BMI-associated SNPs showed, when plotted, a clear change in mean squared error (MSE) around 10 % (Supplementary figure 1a). SNPs above 10 % MSE were considered having a relevant influence on the model and were chosen for inclusion in the genetic risk score. The change in MSE was also seen in the random forest results for the weighted WHR SNPs, although not as clear. The cut off for 10 % was therefore used in that model as well. The trajectory direction for each SNP with a MSE >10 % (i.e., if the SNP increased or decreased EBMIL) was tested by performing a preliminary linear regression analysis. The coding of a BMI or WHR-associated SNP was inverted in case an association with EBMIL increase was detected.

GRS were calculated by summing up the weighted SNP scores for all variants that induced a MSE >10 % in the random forest model according to the following formula: ∑*n* effect alleles_SNPi_ *×* beta value_SNPi_ (*i* = number of included SNPs in the model, *n* = number of risk alleles). The GRSs were used as continuous variables in a multiple linear regression with EBMIL as the dependent variable, adjusting for age, sex, initial BMI, and surgery type. Student’s *t* test was used in the post hoc analysis to compare the strength of weight loss between GRS quartiles. *P* values <0.05 were considered significant and, if necessary, adjusted for multiple testing according to Benjamini-Hochberg (BH) with the Q-level of 5 % [20]. Analyses were performed using the CRAN package “Rattle” (Williams G. Data Mining with Rattle and R: The Art of Excavating Data for Knowledge Discovery.; 2011) in R studio (R Core Team, Vienna, Austria) and SPSS Statistics for Windows, Version 22.0 (IBM Corp. Released 2013. IBM. Armonk, NY: IBM Corp.).

## Results

### Clinical Outcome

Patients showed an average EBMIL of 84 % (SD 18 %) 2 years after RYGB surgery. Younger patients and patients with lower initial BMI benefitted most from the intervention according to regression analyses correcting for age, BMI, sex, and surgery type (*β* = −0.23, *P* < 0.031 resp. *β* = −0.73, *P* < 0.001). Men and women showed a similar EBMIL. The surgery type did not have a significant effect on EBMIL in this cohort. However, we observed a significant difference in the average percentage of individual BMI loss 2 years after RYGB surgery in dependency of proximal or distal surgery (*P* = 0.045). The percentage loss in BMI was stronger in individuals undergoing distal RYGB surgery (33.1 %) compared to patients who underwent the proximal surgery approach (36.7 %).

### Outcome of Random Forest Analyses

Two random forest models were developed to evaluate the impact of genetic variants on EBMIL. In the first model 23 BMI-associated SNPs were included (RFM_wBMI_) [8]. The second model considered 12 WHR-associated SNPs (RFM_wWHR_) [6]. As shown in Table 2, seven variants associated with BMI and three variants associated with WHR induced a >10 % MSE and were considered in the subsequent calculation of GRS scores.

**Table 2:** Genetic variances included in GRS models

	SNP	Gene	Minor allele	Reported effect allele	Risk allele/weight loss lowering allele	MSE (%)
GRS_wBMI_, weighted BMI	rs571312	*MC4R*	A	A	C	23.16
	rs3810291	*TMEM160*	G	A	G	19.65
	rs1555543	*PTBP2*	A	C	A	18.58
	rs206936	*NUDT3*	G	G	G	17.58
	rs987237	*TFAP2B*	G	G	G	17.49
	rs4836133	*ZNF608*	A	A	A	17.12
	rs2241423	*MAP2K5*	A	G	G	13.10
GRS_wWHR_, weighted WHR	rs1443512	*HOXC13*	A	A	A	13.15
	rs4846567	*LYPLAL1*	A	G	G	11.24
	rs1011731	*DNM3*-*PIGC*	G	G	A	10.32

The listed single nucleotide polymorphisms (SNP) induced a mean squared error (MSE) above 10 % in the random forest model. These variants were subsequently included in the respective GRS model (weighted BMI model (GRS_wBMI_) and weighted waist-hip ratio model (GRS_wWHR_)). Those alleles were defined as risk alleles that contributed least to the weight loss following Roux-en Y gastric bypass surgery. Interestingly, the A allele of the TMEM160-related SNP rs3810291 was reported to be associated with higher BMI by Speliotes et al. In our study, the allele, G, is associated with EBMIL

### Impact of GRSs on EBMIL After RYGB Surgery

**Fig. 1:**
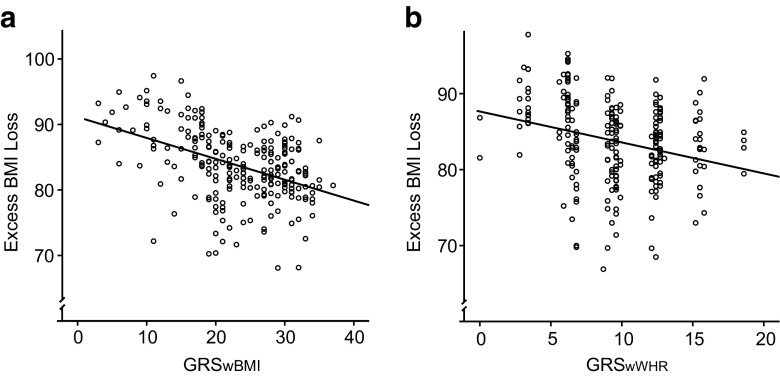
The genetic risk scores (GRS), calculated on the basis of weighted single nucleotide polymorphisms associated with BMI (**a** GRS_wBMI_) and waist-hip ratio (**b** GRS_wWHR_) estimated an up to 11 % difference in excess BMI loss (EBMIL) after Roux-en Y gastric bypass surgery with increasing risk score for both models. Each GRS was inserted individually in an adjusted multiple linear regression with EBMIL as the independent variable. **a** GRS_wBMI_ included genetic variants of *MC4R*, *TMEM160*, *PTBP2*, *NUDT3*, *TFAP2B*, *ZNF608*, *MAP2K5*, *GNPDA2*, and *MTCH2* and had a significant impact on EBMIL in a multilinear regression model adjusting for age, sex, initial BMI, and surgery type (*P* value of 0.026, line equation; *y* = 1.1–0.32*x*; *R* ^2^ = 0.181. **b** GRS_wWHR_ consisted of variants within or near by the genes *HOXC13*, *LYPLAL1*, and *DNM3*-*PIGC*, *P* = 0.021, line equation; *y* = 89.4–0.59*x*; *R* ^2^ = 0.071

The first model, RFM_wBMI_, consisting of BMI-associated SNPs, generated seven SNPs that were included in the BMI SNP-based GRS (GRS_wBMI_, closest genes: *MC4R*, *TMEM160*, *PTBP2*, *NUDT3*, *TFAP2B*, *ZNF608*, *MAP2K5*, *GNPDA2*, and *MTCH2*). The included patients showed GRS_wBMI_ ranging from 3 to 37, thereby carrying at least one risk allele and up to one risk allele in each of the seven genes. The GRS_wBMI_ was significantly associated with EBMIL (*β* = −0.32, *P* = 0.026) in the multiple linear regression model adjusting for age, sex, initial BMI, and surgery type, indicating a 0.32 % decrease of EBMIL per score unit. Maximum and minimum GRS_wBMI_ scores were associated with 83 and 89 % EBMIL, respectively (Fig. 1a). The results were confirmed in linear regression analyses showing a significant impact of GRS_wBMI_ on percent BMI loss (*β* = −0.14, *P* = 0.034). Investigated SNPs and associated genes are shown in Table 2.

RFM_wWHR_ detected three weighted WHR-associated SNPs (closest genes: *HOXC13*, *LYPLAL1*, and *DNM3*-*PIGC*) inducing a 10 % MSE >10 % that were included into a GRS composed of WHR-associated SNPs (GRS_wWHR_). The generated GRS_wWHR_ ranged from 0 to 19 and was associated with EBMIL using multiple linear regression analysis (*β* = −0.59, *P* = 0.021). Maximum and minimum GRS_wWHR_ scores were associated with 78 and 89 % EBMIL, respectively (Fig. 1b). The findings were confirmed in regression analyses showing an association between GRS_wWHR_ and percent BMI loss (*β* = −0.29, *P* = 0.028).

### Quartile Analysis of the GRSs

**Fig. 2:**
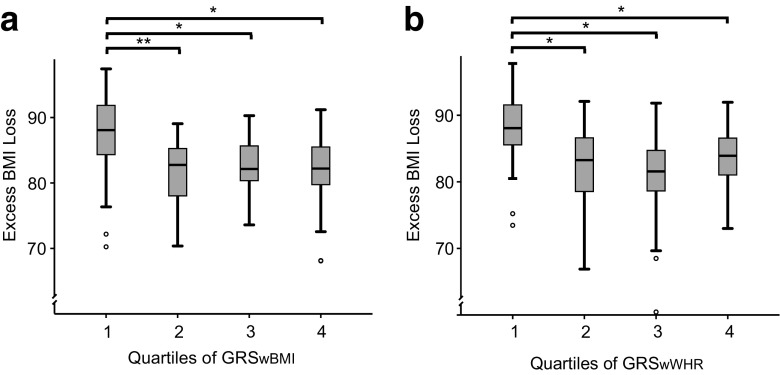
Quartiles of the genetic risk scores (GRS) indicate that patients with none or very few risk alleles have a higher expected excess BMI loss (EBMIL) following RYGB surgery than patients with a higher number of risk alleles. GRSs quartiles were inserted as factors in an adjusted multiple linear regressions with EBMIL as the independent variable. **a** In GRS_wBMI_ Q1, the mean EBMIL was with 89 % significantly different from the means of Q2, Q3, and Q4 (79 to 83 %), Benjamini Hochberg-adjusted *P* < 0.027. **b** GRS_wWHR_ Q1 corresponded to a mean EBMIL of 89 % compared to 81 to 82 % in Q2–Q4 (Benjamini Hochberg-adjusted *P* < 0.048). *P < 0.05, **P < 0.01

To further analyze associations between the genetic composition and post-operative EBMIL, patients were divided into four quartiles based on their GRS. The score quartiles of GRS_wBMI_ and GRS_wWHR_ were entered as a factor in two independent multiple linear regression analyses, correcting for age, sex, initial BMI, and surgery type. In both models, the lowest GRS quartile was associated with a significantly higher EBMIL in contrast to the other quartiles (GRS_wBMI_: BH-adjusted *P* < 0.027, GRS_wWHR_: BH-adjusted *P* < 0.048; Fig. 2a, b). Similar results were obtained when regressing quartiles of the GRSs against percent BMI loss. In contrast to GRS_wWHR_ quartiles, GRS_wBMI_ quartiles showed a significant linear association with percent BMI loss (*P* = 0.007 resp. *P* = 0.051). Further, patients in the first quartile lost more total BMI compared to the other quartiles (GRS_wBMI_: BH-adjusted *P* < 0.021, GRS_wWHR_: BH-adjusted *P* < 0.032).

## Discussion

In the present study, we demonstrate that weight loss (EBMIL) 2 years after RYGB surgery is inversely associated with two genetic risk scores composed of BMI and WHR-associated SNPs. This is the first study that developed genetic risk scores to investigate the impact of common BMI and WHR-associated SNPs on post-operative EBMIL after RYGB surgery using the random forest method as a novel selection approach for relevant SNPs. The EBMIL was up to 11 % different in dependency of SNP composition in both the BMI-associated and the WHR-associated model. These findings suggest a relevant and additive effect of genetic variants on RYGB, translating into about 1.9 kg/m^2^ average weight loss. Speliotes et al. [8] reported a per-allele impact of 0.17 kg/m^2^ for the strongest BMI-associated SNP, *FTO*. Our observations strengthen the hypothesis that the consideration of several risk SNPs helps to estimate post-operative weight response more accurately than isolated genetic variants and can be considered as a complement to the predictive clinical factors such as age and initial BMI.

Patients with the lowest genetic risk scores (in quartile 1; Q1) had a significantly higher EBMIL, compared to higher scoring patients in quartiles 2 to 4 (Fig. 2a, b). This observation supports the hypothesis that patients carrying none or only a small number of the risk alleles show a more efficient weight loss following RYGB surgery than carriers of multiple risk variants. Interestingly, increasing GRS do not seem to interfere proportionally with EBMIL, as the impact of GRS quartiles 2, 3, and 4 on EBMIL did not differ significantly (Fig. 2). This observation indicates that the risk alleles do not follow a strictly linear effect pattern. All tests were also performed for percent BMI loss as the independent variable and led to similar results.

Earlier studies showed that the type of surgery may have a significant impact on weight loss efficacy [21]. Therefore, we included the type of surgery as important covariate in our regression models, when evaluating the genetic risk scores that we developed. While we did not observe significant differences in EBMIL dependent on the surgery type, we did observe a significant better outcome in percentage BMI change 2 years after distal surgery. These results suggest that distal surgery may induce an efficient individual effect on weight loss, from which especially strongly obese patients may profit, despite the fact that the overall rate in EBMIL appears not to be significantly different in proximal and distal surgery.

Not all previously reported effect alleles linked to BMI and WHR in GWAS correspond to the effect allele reported in this study (Table 2). The observed differences in effect may be attributed to the difference in the investigated trait, i.e., BMI investigated in other studies compared to EBMIL investigated in our study. Furthermore, external factors, such as exercise, may impact the strength of association between SNP and trait as, e.g., observed for the gene *FTO* [22].

### Potential Mechanisms

We report that a combination of risk alleles is linked to weight loss following RYGB surgery. When analyzing GRS_wBMI_ and GRS_wWHR_-associated genes with the online tools g:Profiler [23] or String [24] for pathway analysis (and all originally included SNP-associated genes as background), no common known biological pathways were detected, suggesting that weight regulation is impacted via several not directly interfering signaling routes. All genes included in the developed GRS have previously been linked to higher BMI or WHR [6, 8]. Furthermore, we and others could show that three of these genes, namely *MC4R*, *TFAP2B*, and *LYPLAL1*, are associated with weight loss [25–28].

Melanocortin receptor 4 (*MC4R*) has been implicated in eating regulation and metabolism. The protein responds to the agonists alpha- and beta-MSH, which is antagonized by agouti gene-related peptide in the hypothalamus regulating energy intake [29]. Studies investigating the impact of common *MC4R* SNPs on bariatric surgery outcome led to contradictory results [30–33]. Activating enhancer binding protein 2 beta (*TFAP2B*) and lysophospholipase-like 1 (*LYPLAL1*) have not been linked yet to post-surgery weight loss. However, in a meal intervention study, the *TFAP2B*-associated SNP rs987237 was linked to weight loss in the high-fat diet group [27]. *LYPLAL1* is expressed in regions known to regulate energy metabolism such as the hypothalamus and brain stem [34–36]. *LYPLAL1* was associated with weight loss effects in obese patients undergoing lifestyle changes [28]. The SNP rs4846567 close to *LYPLAL1* is located within an insulator segment, which may interfere with the promoter-enhancer interaction and/or block the propagation of heterochromatic structures and DNA-methylation in adjacent chromatin [37]. It can be speculated that the SNP leads to a less available promoter region and consecutively to an altered expression.

### Strengths and Limitations

Previous studies that aimed to define a genetic risk score for weight loss following RYGB did not lead to significant results. These studies summed all putative risk alleles to a GRS without applying a filtering step that allows identifying genetic variants with high impact on the investigated trait [1, 38]. Our approach using the random forest method to detect SNPs with high impact on the outcome variable appears to be an efficient and sensitive way to select relevant SNPs for the subsequently performed association analysis. It would be of value to confirm the obtained results in a larger cohort of RYGB patients.

## Conclusions

Using random forest analysis as a SNP selection tool, we defined two GRSs composed of weighted BMI and WHR-associated SNPs, respectively, which are significantly associated with EBMIL after RYGB surgery. A pre-surgical determination of the GRS will help to pre-surgically evaluate to what extent patients may profit from RYGB surgery.

*BMI*, body mass index; *DNM3*, dynamin 3-phosphatidylinositol glycan anchor biosynthesis, class c; *EBMIL*, excess BMI loss; *GNPDA2*, glucosamine-6-phosphate deaminase 2; *GRS*, genetic risk score; *GWAS*, genome wide association study; *HOXC13*, homeobox c13; *LYPLAL1*, lysophospholipase-like 1; *MAP2K5*, mitogen-activated protein kinase kinase 5; *MC4R*, melanocortin 4 receptor; *MSE*, mean standard error; *MTCH2*, mitochondrial carrier 2; *NUDT3*, nudix-type motif 3; *PTBP2*, polypyrimidine tract binding protein 2; *RFM*, random forest model; *RYGB*, Roux-en Y gastric bypass; *SNP*, single nucleotide polymorphism; *TFAP2B*, activating enhancer binding protein 2 beta; *TMEM160*, transmembrane protein 160; *WHR*, waist-hip ratio; *ZNF608*, zinc finger protein 608.

## Electronic Supplementary Material

Below is the link to the electronic supplementary material.

Supplementary table 1 (XLSX 19 kb)

Supplementary figure 1 The mean squared error introduced by each SNP in the random forest models. In a) all BMI-associated SNPs were included and in b) all WHR-associated SNP were included. The vertical dashed line indicates the cut off for inclusion into the GRS models (10 %). (GIF 2 kb)

High Resolution Image (TIF 84 kb)
