# Supplementary figures and images for: A Genetic Risk Score Is Associated with Weight Loss Following Roux-en Y Gastric Bypass Surgery
Source: Obes Surg. 2016 Jan 30;26(9):2183–9. doi: 10.1007/s11695-016-2072-9 (PMC4985537; doi:10.1007/s11695-016-2072-9)

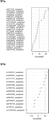

Supplement: Supplementary file 2 — The mean squared error introduced by each SNP in the random forest models. In a) all BMI-associated SNPs were included and in b) all WHR-associated SNP were included. The vertical dashed line indicates the cut off for inclusion into the GRS models (10 %). (GIF 2 kb) [file 11695_2016_2072_Fig3_ESM.gif]
